# Prevalence and Antibiotic Resistance of *Campylobacter* spp. in Urban and Rural Black-Headed Gulls *Chroicocephalus ridibundus*

**DOI:** 10.1007/s10393-021-01540-0

**Published:** 2021-09-03

**Authors:** Piotr Indykiewicz, Małgorzata Andrzejewska, Piotr Minias, Dorota Śpica, Jarosław Kowalski

**Affiliations:** 1 Department of Biology and Animal Environment Landscaping, Bydgoszcz University of Science and Technology, Mazowiecka 28, 85-084 Bydgoszcz, Poland; 2 Department of Hygiene, Epidemiology, Ergonomy and Postgraduate Education, Nicolaus Copernicus University, M. Curie-Skłodowskiej 9, 85-094 Bydgoszcz, Poland; 3 Department of Biodiversity Studies and Bioeducation, Faculty of Biology and Environmental Protection, University of Łódź, Banacha 1/3, 90-237 Łódź, Poland; 4 Gorzewo 7, 09-200 Sierpc, Poland

**Keywords:** Antibiotic resistance, Birds, Black-headed gull, Campylobacteriosis, Urbanization, Zoonoses

## Abstract

We investigate the role of black-headed gulls (*Chroicocephalus ridibundus*), an omnivorous species that is among the most likely wild bird candidates for transmission of zoonotic agents, as a potential reservoir of *Campylobacter* spp. Colonies with different anthropogenic pressures were studied to examine differences in exposure to sources of *Campylobacter* between rural and urban birds. We recorded *Campylobacter* spp. in 4.87% of adult black-headed gulls and 2.22% of their chicks after analysing 1036 cloacal swabs collected over two breeding seasons in three colonies in northern Poland. *Campylobacter jejuni* was found most frequently (85.72%), and *Campylobacter lari* and *Campylobacter coli* were much scarcer. Prevalence of *Campylobacter* did not differ significantly between black-headed gulls breeding in urban (4.27%) and rural (3.80%) habitats. Almost all isolates from chicks and adults were susceptible to azithromycin (97.62%) and erythromycin (95.24%), but fewer to tetracycline (50.00%) and ciprofloxacin (47.62%). *Campylobacter* prevalence was unrelated to the date of sampling. Our study indicates that black-headed gulls are carriers of resistant to antibiotics *Campylobacter* and they can contaminate natural waterbodies with their faeces, which poses a threat to human and farm animal health.

## Introduction

*Campylobacter* and *Salmonella* spp. are leading causes of zoonotic stomach and intestinal infections in humans, both in developing and developed countries, and the frequency of these infections is increasing even in countries with efficient public healthcare systems (WHO 1980, Oberhelman and Taylor 2000; Friedman et al. 2001; Baker et al. 2007; Olson et al. 2008). Increasing prevalence of campylobacteriosis in South America, Europe and Australia is alarming, and data from Africa, Asia and the Middle East show that it is endemic in these areas, especially among children (Kaakoush et al. 2015). The European Food Safety Authority (EFSA) and the European Centre for Disease Prevention and Control (ECDC) report that campylobacteriosis affects up to 246 000 residents of the European Union (EU) per year (EFSA 2018), but the real number of annual *Campylobacter* infections is estimated as ca 9 million a year (EFSA 2011a, Havelaar et al. 2009). Since 2005, the total number of campylobacteriosis cases in the EU has exceeded the number of reported salmonellosis cases, and it is still rising in some EU countries (EFSA 2011b, 2018). Campylobacteriosis affects people of all ages, but infections are most frequent in children younger than five (Kaakoush et al. 2015) and city dwellers older than 50 (Nichols et al. 2012). Workers at abattoirs, mainly of poultry, as well as veterinarians, breeders of poultry, cattle and pigs, and ornithologists, are especially exposed to infections (Jones 2001; Abulreesh et al. 2007; Humphrey et al. 2007; Silva et al. 2011; WHO 2013). Two autoimmune neurological disorders, Guillain–Barre and Miller-Fisher syndromes, have been associated with *C. jejuni* infections (Ang et al. 2001; van Doorn et al. 2008). The EFSA has estimated that *Campylobacter* infections cause work absences in the EU costing about €2.4 billion a year (EFSA 2011a,b) and in the USA about $1.6 billion (Scharff 2011). Taking all this into account, campylobacteriosis is considered one of the most widespread and important infectious diseases that poses an increasing threat to global health (Kaakoush et al. 2015).

Despite the threat to human health, *Campylobacter* epidemiology is not yet fully understood, and epidemiological pathways leading to humans are incompletely recognized (Ramos et al. 2010; Kaakoush et al. 2015). We also do not know much about *Campylobacter* infections in wild animals, such as the duration of infection, if *Campylobacter* produce clinical or subclinical patterns, or if infected animals gain temporary or general immunity (Broman et al. 2002). *Campylobacter* are widely distributed in nature, and the main risk factors for humans are international travel; food of animal origin (especially milk and poultry—Hänninen et al. 2003; Baker et al. 2006; Gu et al. 2009; Acke et al. 2011; Kaakoush et al. 2015; EFSA, 2017); direct contact with animal hosts and natural waters polluted by bird faeces (Humphrey et al. 2007; Kaakoush et al. 2015). The digestive systems of wild and domesticated birds and mammals are the main reservoirs of *Campylobacter*. Extensive evidence exists of wild birds being directly responsible for causes of zoonotic gastrointestinal infections in humans (Gardner et al. 2011; Rutledge et al. 2013; Kaakoush et al. 2015).

Gulls are among birds frequently tested for public health reasons, because they often forage at rubbish dumps or near inlets to sewage plants (Smith and Carlile 1993a; Hatch 1996; Belant 1997; Clark et al. 2013; Egunez et al. 2018). Also, population sizes of many gull species in Europe, North America and Australia have increased considerably over the past few decades (Smith and Carlile 1993b; Vidal et al. 1998; Perriman and Lalas 2012; Washburn et al. 2016), which may pose an increasing epidemiological threat.

Our study aimed to assess *Campylobacter* prevalence in the black-headed gull *Chroicocephalus ridibundus*, a common species associated mainly with inland freshwater habitats*.* Black-headed gulls are migratory and long-lived (van Dijk et al. 2012), and they also exhibit strong philopatry (Peron et al. 2010) and may return to the same colonies over many years, even when breeding habitats undergo unfavourable changes (Burger 1979). The black-headed gull is an omnivorous and opportunistic species, which searches for food in natural habitats, such as seacoasts and the banks of lakes and rivers, in arable areas and urban habitats. Here, we specifically aimed to determine: (i) prevalence of *Campylobacter* spp. in the Polish population of black-headed gulls in association with age and type of habitat (urban versus rural), (ii) prevalence of *Campylobacter* in the social partners and offspring of infected individuals and (iii) resistance of recorded *Campylobacter* isolates to selected antibiotics.

## Material and Methods

### Study Site and General Field Procedures

We studied black-headed gulls in three breeding colonies located in north-central Poland. Two urban colonies were situated ca 15.5 km apart on the opposite sides of Bydgoszcz city (ca. 400 000 inhabitants). One site was located in an industrial zone (Bydg-IND), the other in a recreational area (Bydg-REC). The Bydg-IND colony was located on a small island (53°7′7.9" N, 18°6′19.1" E) in a natural bay of the Brda River. The Bydg-REC colony (53°9′51.5" N, 18°2′12.1" E) occurred in a small reservoir abutting the zoological garden and a vast recreational area. The third (rural) colony was located on an island at Kusowo lake (53°15′0.3" N, 18°8′28.8" E), a natural reservoir surrounded by farmland. In 2015–2016, 330–350 pairs of black-headed gulls bred in the Bydg-IND colony, 120 pairs in Bydg-REC and 880–1200 pairs in Kusowo. The extent of anthropogenic pressure, physiographic characteristics of the areas surrounding the breeding sites and detailed information about the study area were described by Kitowski et al. (2017) and Indykiewicz et al. (2018a).

For the purpose of *Campylobacter* detection, we collected cloacal swabs from 718 adult (older than 2 years) black-headed gulls and 318 of their chicks aged 7–21 days. All captured birds were individually marked to avoid repeated sampling of the same individuals. Sample sizes were similar for urban (*n* = 473 swabs) and rural (*n* = 563 swabs) colonies (Table 1). All samples were collected between 19 April and 16 June. The median sampling dates were 12 May and 06 June for adults and chicks, respectively. The total number of sampling events was 31 (16 in 2015 and 15 in 2016) for Bydg-IND, 11 (only 2016) for Bydg-REC and 38 (12 in 2015 and 26 in 2016) for Kusowo. Samples were stored in sterile Amies charcoal transport medium (Transwab® ENT Amies) in a portable fridge at 1–4 °C. Within 4h from collection, we brought the swabs to the laboratory, where they immediately underwent the isolation procedure.

**Table 1:** PCR Primers used for *Campylobacter* Detection

Species	Primer	Sequence (5′ to 3′)
Genus *Campylobacter*	C412F	5′-GGATGACACTTTTCGGAGC-3′
	C1228R	5′-CATTGTAGCACGTGTGTC-3′
*C. hyointestinalis*	HYO1F	5′-ATAATCTAGGTGAGAATCCTAG-3′
	HYOFET23SR	5′-GCTTCGCATAGCTAACAT-3′
*C. coli*	CC18F	5′-GGTATGATTTCTACAAAGCGAG-3′
	CC519R	5′-ATAAAAGACTATCGTCGCGTG-3′
*C. fetus*	MG3F	5′-GGTAGCCGCAGCTGCTAAGAT-3′
	CF359R	5′-AGCCAGTAACGCATATTATAGTAG-3′
*C. lari*	CLF	5′-TAGAGAGATAGCAAAAGAGA-3′
	CLR	5′-TACACATAATAATCCCACCC-3′
*C. jejuni*	C-1	5′-CAAATAAAGTTAGAGGTAGAATGT-3′
	C-3	5′-CCATAAGCACTAGCTAGCTGAT-3′
*C. upsaliensis*	CU61F	5′-CGATGATGTGCAAATTGAAGC-3′
	CU146R	5′-TTCTAGCCCCTTGCTTGATG-3′

### Campylobacter Isolation and Identification

Isolation of *Campylobacter* bacteria was based on the method described previously by French et al. (2009), in which the material requires initial incubation in 3 ml of Bolton broth (Oxoid) in conditions with reduced oxygen supply (85% N_2_, 10% CO_2_, 5% O_2_—Generbox microaer, BioMerieux) in temperature 42 °C for 48 h. Next 10 μl of the enrichment culture was streaked onto agar plates (Charcoal Cefoperazone Desoxycholate Agar plates—mCCDA, Oxoid) under the conditions described above. Grown colonies were evaluated for morphology and subject to biochemical tests. The catalase and oxidase reactions were performed using available tests (Oxoid). The colonies recognized as presumptive *Campylobacter* spp. were retained for further study.

Confirmation of isolates to *Campylobacter* genus and species identification was conducted with multiplex PCR. The presence of *Campylobacter* target genes was determined with primers set together in Table 1. The conditions of DNA amplification reaction used in our study were described by Yamazaki-Matsune et al. (2007). Bacterial DNA was isolated from cultures grown on Columbia agar with 5% of sheep blood after microaerophilic incubation for 24 h (de Lamballerie et al. 1992). PCR amplification was performed in a mixture of 5 μl of DreamTaq PCR Buffer (Fermentas), 0.5 μl of dNTPs (10 mM Fermentas), 1 μl of each primer (1 μM), 1U of Dream Taq DNA polymerase (Fermentas) and 1 μl of template DNA, and then, the final volume was adjusted to 25 μl.

We performed DNA amplification in a Tpersonal thermal cycler (Biometra) and then analysed PCR products by electrophoresis in 1.5% agarose gel. DNA bands were visualised by staining the samples with Midori Green DNA Stain (Nippon Genetics) and photographed using the IG/LE InGenius L documentation system (Syngene). PCR amplicons were compared with DNA length markers (Fermentas).

### Antimicrobial Susceptibility Testing

We determined the susceptibility of *Campylobacter* isolates to clinically important antimicrobials: erythromycin, azithromycin, tetracycline and ciprofloxacin. E-test (AB Biodisk) was applied on Mueller–Hinton agar supplemented with 5% defibrinated horse blood (Oxoid). The plates, inoculated with test strain, were incubated at 37 °C for 48 h under microaerophilic conditions. MIC values were determined according to Clinical and Laboratory Standards Institute’s guidelines for the *Enterobacteriaceae* family (CLSI 2008). Thresholds for *Campylobacter* resistance were set as follows: erythromycin 32 µg/mL, tetracycline 16 µg/mL, azithromycin 8 µg/mL and ciprofloxacin 4 µg/mL. We used *C*. *jejuni* ATCC 33,560 and *C*. *coli* ATTC 33,559 as reference strains.

### Statistical Analyses

Prevalence of *Campylobacter* in black-headed gulls was analysed with generalized linear models (GLMs) for the binomial distribution of a response variable (0—no *Campylobacter* recorded, 1—*Campylobacter* recorded). The effect of colony location (urban vs. rural) was tested separately for adults and nestlings. To test for the effect of year, we used data exclusively from adult birds (sampled in both years, 2015–2016), while to test or the effect of age (adult *vs.* nestling), we used data exclusively from 2016 (both ages sampled). Sampling date was entered as a covariate in each model. Similar models were run for the occurrence of resistant *Campylobacter* isolates (0—non-resistant strain, 1—resistant strain). All models were run using the *lme4* package (Bates et al. 2015) for R (R Development Core Team 2013). We used the *car* package (Fox and Weisberg 2011) to determine Wald Chi^2^ (W) statistics and P values for all predictors.

## Results

We detected *Campylobacter* spp. in 35 of 718 (4.87%) samples from adult black-headed gulls and in 7 of 318 (2.22%) samples from chicks (Table 2). We found no significant difference in *Campylobacter* prevalence between the two age categories (*W* = 0.04, *df* = 1, *P* = 0.84). At the age of 1–2 weeks, there were five chicks positive for *Campylobacter*, and at the age over 2 weeks—only two individuals. *C. jejuni* was most frequently identified in positive samples (85.72%. *n* = 42), whereas *C. lari* and *C. coli* were recorded significantly less frequently (7.14% each). Prevalence of *Campylobacter* in black-headed gulls from urban and rural habitats did not differ significantly, neither in adults (4.56% *vs* 5.20%; *W* =  0.02, *df* = 1, *P* = 0.88) nor nestlings (0.99% *vs* 2.77%; *W* = 1.77, *df* = 1, *P* = 0.18). However, we found that *Campylobacter* prevalence in adult gulls differed significantly between the two breeding seasons (*W* = 4.12, *df* = 1, *P* = 0.042). Specifically, prevalence was higher in 2015 than in 2016, and this difference occurred in both study colonies that were sampled in both seasons (Kusowo: 6.74% and 3.59% in 2015 and 2016, respectively; Bydg-IND: 7.14% and 2.04% in 2015 and 2016, respectively). We found no support for an association between *Campylobacter* prevalence and sampling date in adults (*W* =  0.11, *df* = 1, *P* = 0.74), but in nestlings, this relationship was only marginally non-significant (*W* =  3.32, *df* = 1, *P* = 0.068), indicating a trend for a decreasing prevalence with date (β = -0.059 ± 0.029).

**Table 2:** Prevalence of different *Campylobacter* Species in Cloacal Swabs Collected from Adult (*ad.*) and Chick (*pull.*) Black-headed Gulls in 2015–2016 at Urban (U) and Rural (R) Habitats in Northern Poland.

Year	Breeding colony	N samples		*C. jejuni*		*C. lari*		*C. coli*	
		*ad*	*pull*	*ad*	*pull*	*ad*	*pull*	*ad*	*pull*
2015	Kusowo (R)	179	–	10	–	2	–	–	–
	Bydg-IND (U)	132	–	9	–	–	–	–	–
2016	Kusowo (R)	167	217	5	5	–	–	1	1
	Bydg-IND (U)	146	101	3	–	–	–	–	1
	Bydg-REC (U)	94	–	4	–	1	–	–	–
	TOTAL	718	318	31	5	3	–	1	2

Out of 35 adults positive for *Campylobacter*, 27 individuals had their social partners also examined for *Campylobacter* occurrence. We found no simultaneous infection with these bacteria in both partners in any pair. Almost all birds (97.1%) in which we found *Campylobacter* in rural habitats bred at the periphery of their breeding colony; only one infected gull nested in the centre of a colony.

Almost all *Campylobacter* isolates from black-headed gulls were susceptible to azithromycin (97.62%) and erythromycin (95.24%). Half of isolates were resistant to tetracycline (50.00%) and ciprofloxacin (47.62%) (Table 3). Almost one-third of *Campylobacter* isolates were resistant to two antibiotics (ciprofloxacin and tetracycline—12/42, or erythromycin and tetracycline—2/42), and, exceptionally, to three antibiotics (azithromycin, erythromycin and tetracycline—1/42). Isolates resistant to tetracycline occurred more frequently in adults than chicks (*W* =  4.01, *df* = 1, *P* = 0.045), but we found no such difference in the *Campylobacter* resistant to ciprofloxacin (*W* =  0.65, *df* = 1, *P* = 0.42). Similarly, the proportion of isolates resistant to tetracycline was significantly higher in 2015 than in 2016 (61.9% *vs* 21.4%; *W* =  6.37, *df* = 1, *P* = 0.011), but we found no such difference for ciprofloxacin (*W* =  0.40, *df* = 1, *P* = 0.53). The proportion of isolates resistant to tetracycline and ciprofloxacin did not differ between adult black-headed gulls from urban and rural colonies (tetracycline: *W* =  0.19, *df* = 1, *P* = 0.66; ciprofloxacin: *W* =  0.13, *df* = 1, *P* = 0.72). Also, we found no association between prevalence of *Campylobacter* isolates resistant to these antibiotics and sampling date (tetracycline: *W* =  1.19, *df* = 1, *P* = 0.28; ciprofloxacin: *W* =  0.01, *df* = 1, *P* = 0.92).

**Table 3:** Antibiotic resistance of *Campylobacter* Strains Isolated from Cloacal Swabs of Black-headed Gull Chicks and Adults in in Northern Poland 2015–2016.

*Campylobacter* (N of isolates)	Azithromycin		Erythromycin		Tetracycline		Ciprofloxacin	
	N	%	N	%	N	%	N	%
*C. jejuni* (36)	0	0.00	1	2.77	22	61.11	20	55.55
*C. lari* (4)	0	0.00	0	0,00	1	25.00	2	50.00
*C. coli* (2)	1	50.00	1	50.00	2	100.00	1	50.00

## Discussion

Current knowledge on the prevalence of *Campylobacter* in black-headed gulls is mostly based on material collected during winter or migration (Kapperud and Rosef 1983; Broman et al. 2002; Moore et al. 2002; Cody, 2015). As far as we are aware, adults and chicks from the same breeding colonies have never been studied. Similarly, studies of black-headed gulls breeding across urbanization gradient are also lacking. Here, we documented prevalence of *Campylobacter* spp. in chicks (2.2%) and adults (4.9%) from the same colonies in northern Poland. Also, for the first time, we compared *Campylobacter* prevalence in adult black-headed gulls from colonies located within and outside urban areas. *Campylobacter* prevalence in our study populations was similar to that recorded in post-breeding season in the Great Britain (2.1–3.5%—Cody et al. 2015) and Northern Ireland (7.1%—Moore et al. 2002), but was lower than in Sweden (13.2–42.9%—Kapperud and Rosef 1983; 27.9–36.2%—Broman et al. 2002) and Czech Republic (63%—Sixl et al. 1997). In our study, we found similar prevalence of *Campylobacter* between adults and chicks, which is no surprising taking into account feeding method—food is regurgitated by the adults for the chicks to eat. Horizontal transmission between chicks within the breeding or between chicks from different nests when they are older and free to move within the colony cannot be ruled out. On the other hands, we recorded no cases of horizontal transmission of *Campylobacter* between social mates. It could be explained by the fact that pairs only physically interact during copulation (which is very brief and usually lasts for a short time, mainly in the pre-laying period). Lack of horizontal transmission may suggest that *Campylobacter* is more easily transmitted from adults to offspring rather than between adults, but further research is needed to provide more empirical support for this hypothesis.

The number of studies on the prevalence of *Campylobacter* spp. in gulls chicks is limited. Infection of broiler flocks by *C. jejuni* usually starts from the third week and increases with age (Hermans et al. 2011; Sahin et al. 2003). In our study, out of seven positive for *Campylobacter* chicks, five were at the age of 7 to 14 days, and only two individuals were aged between 16–21 days. This result contradicts previous studies of Sahin et al., (2003) where active immune responses to *Campylobacter* in broilers chicks occurred earlier and more strongly in birds infected at 21 days of age than these infected at 3 days of age. Further studies need to be performed to explain age-related sensitivity to *Campylobacter* colonization in gulls chicks.

Despite our expectations, we found similar prevalence of *Campylobacter* in black-headed gulls breeding in urban (4.3%) and rural (3.8%) habitats. Our previous study has shown that birds from the urban colony in Bydgoszcz forage mainly on the territory of the city and in its immediate vicinity, while birds from non-urban colonies tend to avoid this type of habitat during foraging flights (Jakubas et al. 2020). We predicted that urban birds may be exposed to more sources of *Campylobacter* than rural birds, e.g. the remains of food in rubbish bins and disposal points near human residences, on municipal rubbish dumps and in sewage works. For example, high proportion of anthropogenic food remains in the diet the yellow-legged gull *Larus michahellis* was associated with higher prevalence of *Campylobacter* spp. (Ramos et al. 2009). On the other hands, rural gulls that mostly forage in farmland may be more exposed to interactions with domestic and farm animals, which could produce similar levels of *Campylobacter* prevalence in urban and rural habitats. In fact, several studies on humans showed higher campylobacteriosis rate in rural areas than in the cities (Strachan et al. 2009; Lévesque et al. 2013). Further studies, ideally supported by diet composition, are needed to better explain the lack of significant differences in *Campylobacter* prevalence among the three colonies.

While studying black-headed gulls in rural areas (Kusowo), we noted that almost all individuals infected with *Campylobacter* (97.1%) located their nests at the periphery of the breeding colony. This corresponds with results of our earlier studies in the same colony, where we found evidence for higher physiological condition of birds breeding in the colony centre (Indykiewicz et al. 2018b). Thus, it seems likely that central pairs may also be more resistant to pathogens than peripheral low-quality pairs. Finally, individuals with more efficient immune system have a greater chance of occupying a high-quality territory in the centre of the colony, and individuals with lower resistance may deliberately avoid the densities in the centre that should promote higher transmission rate of pathogens.

Intra-annual dynamics of human campylobacteriosis may be primarily associated with the composition of breeding, rather than migratory or wintering, avifauna. Infections in temperate countries of the Northern Hemisphere, such as Wales, Denmark, Finland and Sweden, peak between early May and mid-July week of the year (Nylen et al. 2002; Cody et al. 2015). Prevalence of *Campylobacter* in broiler flocks peaks in the early summer months (Boysen et al. 2011) in contradiction to lambs and dairy cattle which had two peaks per year, in approximately spring and autumn (Stanley et al. 1998a,b), but it coincides with breeding season of most avian species, including black-headed gulls. This might suggest that wildlife plays an important role in spreading *Campylobacter* bacteria to humans, and this hypothesis has received non-negligible empirical support. For example, Cody et al. (2015) suggested that in Oxfordshire county alone wild birds might cause campylobacteriosis in as many as 10 000 humans a year. Despite this evidence, the prevailing opinion is that wild birds play a relatively minor role in the epidemiology of *C. jejuni* infections in humans. The crucial argument to support this thesis is based in the genotype and the serotype differences between the strains of *C. jejuni* isolated from man, broiler chicks, pigs and wild birds (Rosef et al. 1985; Whelan et al. 1988; Petersen et al. 2001; Broman et al. 2002).

Although most people with *C. jejuni* infection can successfully recover without specific medical treatment, in many countries infections are, by default, treated with antibiotics, mostly macrolides in combination with azithromycin or aminoglycosides in severe cases (Moore et al. 2002; Bolinger and Kathariou, 2017). Antibiotics are increasingly dispensed to humans in a prophylactic way, for example, to prevent travel diarrhoea, for which fluoroquinolones and tetracyclines are often prescribed (Guerrant et al. 2001). The common use of antibiotics in human therapy increases the number of *Campylobacter* strains resistant to these medicines (Kaakoush et al. 2015), and this process is further enhanced by veterinary use of antibiotics, where only 20% are administered to cure infections, while the remaining 80% are used prophylactically and to stimulate livestock growth, a practice banned in the EU in 2006 (Chiesa et al. 2015). Tetracyclines, fluoroquinolones, macrolides and sulphonamides are most often used in veterinary practice (EMA, 2016), and a large proportion (10–90%) of those are later excreted by animals into the environment in an unchanged form or as active metabolites (Sturini et al. 2014). Therefore, as much as 44.1% of *C. jejuni* isolates from animals, mostly from poultry, is resistant to ciprofloxacin, and 34.1% of the isolates are resistant to tetracycline in the EU (EFSA 2014). In our study population of black-headed gulls, the resistance to these two antibiotics was slightly higher (55.6% and 51.2%, respectively). In black-headed gulls chicks from Moravia, Czech Republic, all *Campylobacter* isolates (100%) were resistant to tetracycline and to ampicillin (Sixl et al. 1997). *C. jejuni* isolated in Iberian coast from yellow-legged gulls (*Larus michahellis*), resistant to ciprofloxacin (67%) and tetracycline (34%) were previously described by Lourdes et al. (2017). In Jurinović et al.’s study (2020), all *C. jejuni* isolated from gulls in Croatia (yellow-legged gull, black-headed gull, caspian gull—*Larus cachinanns*, herring gull—*L. argentatus* and common gull—*L. canus were*) found to be susceptible to erythromycin, while 2.0% of isolates were found to be resistant to gentamicin and 46% to tetracycline.

Antibiotic resistance of *Campylobacter* strains in other species of wild birds largely varies. For example, a study of the White Stork *Ciconia ciconia* in Poland found that 19.0% and 52.4% of C*. jejuni* isolates were resistant to tetracycline and ciprofloxacin, respectively (Szczepańska et al. 2015). In the long-eared owl, *Asio otus* in Sweden, the resistance to ciprofloxacin was found in 11.1% of isolates (Waldenström et al. 2005), while in the house crow *Corvus splendens* and pigeons from the Serdang region in Malaysia the resistance of *Campylobacter* to tetracycline recorded in 33.3% of isolates (Mustaffa et al. 2014).

In conclusion, the results of our study indicate that black-headed gulls harbour *Campylobacter* spp. More importantly, we confirmed that black-headed gulls are carriers of antibiotic-resistant *Campylobacter*, which may pose a threat to human and farm animal health. We also underline the need of further studies to assess the role of black-head gull in *Campylobacter* spp. epidemiology. Further studies comprising genetic relatedness of *Campylobacter* isolates from human, wild birds and water sources are needed as they can confirm environmental transmission of *Campylobacter* by polluting artificial and natural waterbodies, soil and plants with their faeces.
